# Development of an Improved Cadaveric Training Model for Endoscopic Combined Intrarenal Surgery Using Fresh‐Frozen Cadavers

**DOI:** 10.1111/iju.70671

**Published:** 2026-10-13

**Authors:** Keito Shiozaki, Yutaro Sasaki, Katsuhito Hori, Marika Atagi, Ryota Nakanishi, Keisuke Hashimoto, Koki Tada, Fumiya Kadoriku, Takuya Yoshioka, Kyotaro Fukuta, Yuichiro Atagi, Saki Kobayashi, Ryoei Minato, Ryotaro Tomida, Yoshito Kusuhara, Kunihisa Yamaguchi, Tomoya Fukawa, Hirofumi Izaki, Koichi Tomita, Junya Furukawa

**Affiliations:** ^1^ Department of Urology Tokushima University Graduate School of Biomedical Sciences Tokushima Japan; ^2^ Department of Urology Ehime Prefectural Central Hospital Matsuyama Ehime Japan; ^3^ Department of Urology Takamatsu Red Cross Hospital Takamatsu Japan; ^4^ Department of Urology Chubu Tokushukai Hospital Okinawa Japan; ^5^ Department of Urology Tokushima Prefectural Central Hospital Tokushima Japan; ^6^ Department of Anatomy and Developmental Neurobiology Tokushima University Graduate School of Biomedical Sciences Tokushima Japan

**Keywords:** cadaver surgical training, endoscopic combined intrarenal surgery (ECIRS), nephrostomy puncture, surgical education, surgical simulation

## Abstract

**Objectives:**

Endoscopic combined intrarenal surgery (ECIRS) is technically demanding and has a prolonged learning curve. Reports describing ECIRS training using fresh‐frozen cadavers are limited, partly because intrarenal and pyelovenous backflow may prevent adequate hydronephrosis. This study aimed to establish an optimized ECIRS training model using fresh‐frozen cadavers.

**Methods:**

ECIRS cadaver surgical training (ECIRS‐CST) was conducted between April 2021 and October 2022 and evaluated across two periods. In the first period, standard ECIRS‐CST was performed. In the second period, laparoscopic nephrectomy training with renal vein clipping was performed before ECIRS‐CST to suppress pyelovenous backflow. Achievement of each ECIRS step and overall procedural completion were recorded. Participants assessed procedural realism and educational value using a custom‐designed 5‐point Likert‐scale questionnaire.

**Results:**

Nine sessions were conducted in the first period and four in the second. During the first period, ultrasound‐guided nephrostomy puncture frequently failed because adequate hydronephrosis could not be established, and only three sessions progressed through all ECIRS steps. In the second period, renal vein clipping enabled more consistent hydronephrosis, resulting in successful nephrostomy puncture and completion of all steps in three of four sessions. Questionnaire responses indicated that tactile feedback during puncture, ultrasonographic visualization, and endoscopic findings were comparable to those in live patients. Many participants reported difficulty in achieving sufficient hydronephrosis during the first period.

**Conclusions:**

Fresh‐frozen cadavers provide a realistic environment for ECIRS training; however, pyelovenous backflow limits the creation of adequate hydronephrosis. Renal vein clipping effectively addresses this limitation and provides a more reliable training model for procedural training.

## 1 Introduction

Endoscopic combined intrarenal surgery (ECIRS) is widely used to treat complex renal calculi, including staghorn and large stones. By combining retrograde intrarenal surgery with percutaneous nephrolithotomy, ECIRS achieves high stone‐free rates with acceptable safety. However, it requires advanced endourological skills and multiple procedural steps, resulting in a steep learning curve [1, 2].

Among these steps, nephrostomy puncture is a key determinant of procedural success. Accurate puncture affects operative time, stone‐free rates, and complication risk. In practice, nephrostomy puncture during ECIRS is often performed by only a few operators, even in high‐volume centers, limiting trainees' opportunities to gain experience. Moreover, essential aspects of puncture technique—such as the tactile sensation of penetrating the renal capsule and pelvis, puncture angle, and puncture speed—are difficult to master without hands‐on training.

Cadaver surgical training (CST) provides a realistic, safe environment without exposing patients to risk. A single CST session enables multiple urologists to practice nephrostomy puncture under conditions that approximate live surgery. Prior studies have demonstrated the value of CST for endourological procedures using Thiel‐embalmed cadavers [3]. However, Thiel embalming has limitations, including altered tissue elasticity and incomplete reproduction of procedural sensation [4]. By contrast, fresh‐frozen cadavers more closely preserve tissue texture and anatomical relationships, potentially offering a more realistic model for endourological training [5, 6].

Despite these advantages, ECIRS training with fresh‐frozen cadavers has rarely been described [6]. A major challenge is creating adequate hydronephrosis during retrograde pyelography, which is essential for successful nephrostomy puncture. In fresh‐frozen cadavers, reduced renal venous perfusion pressure permits retrograde flow from the urinary tract into the venous system, limiting pelvic dilatation.

This study evaluated the feasibility of ECIRS training using fresh‐frozen cadavers, identified procedural differences from live‐patient surgery, and developed an improved training model. Specifically, we assessed whether renal vein clipping before ECIRS training facilitates adequate hydronephrosis and improves nephrostomy puncture success.

## 2 Materials and Methods

### 2.1 Study Design and Participants

This educational observational study evaluated the feasibility and effectiveness of CST for ECIRS using fresh‐frozen cadavers. ECIRS‐CST sessions were conducted between April 2021 and October 2022 at the Clinical Anatomy Education and Research Center of Tokushima University Hospital. Nine urologists participated, including two board‐certified urologists and seven urology residents. All had prior ureteroscopy experience but limited experience as primary operators for nephrostomy puncture. However, all participants had previous exposure to nephrostomy puncture procedures under supervision during residency training.

### 2.2 Cadaver Preparation

Fresh‐frozen cadavers donated for medical education were used for all CST sessions. Cadavers were thawed at room temperature before training. No vascular perfusion or artificial circulation was performed. When multiple ECIRS‐CST sessions were conducted using the same cadaver, the left and right kidneys were used separately and counted as independent training units. The two kidneys were generally used during separate training sessions conducted on different days.

### 2.3 ECIRS Training Protocol

#### 2.3.1 First Period (April 2021–March 2022)

During the first period, standard ECIRS‐CST was performed with fresh‐frozen cadavers in the modified Valdivia position. The procedure comprised six steps (Figure 1).

Step 1: A guidewire was inserted into the ureter using a cystoscope (Ambu aScope 4 Cyst) and advanced into the renal pelvis.
Step 2: A ureteral access sheath (UroPass 10/12 Fr or 11/13 Fr; Olympus, Tokyo, Japan) was placed, and the renal pelvis was inspected with a flexible ureteroscope (WiScope).
Step 3: Hydronephrosis was created by retrograde pyelography through the ureteral access sheath.
Step 4: Ultrasound‐guided nephrostomy puncture was performed.
Step 5: The tract was dilated using an Ultraxx Nephrostomy Balloon Catheter, followed by placement of an 18‐Fr nephrostomy sheath.
Step 6: The renal pelvis was inspected with a rigid nephroscope (Olympus).

**FIGURE 1:**
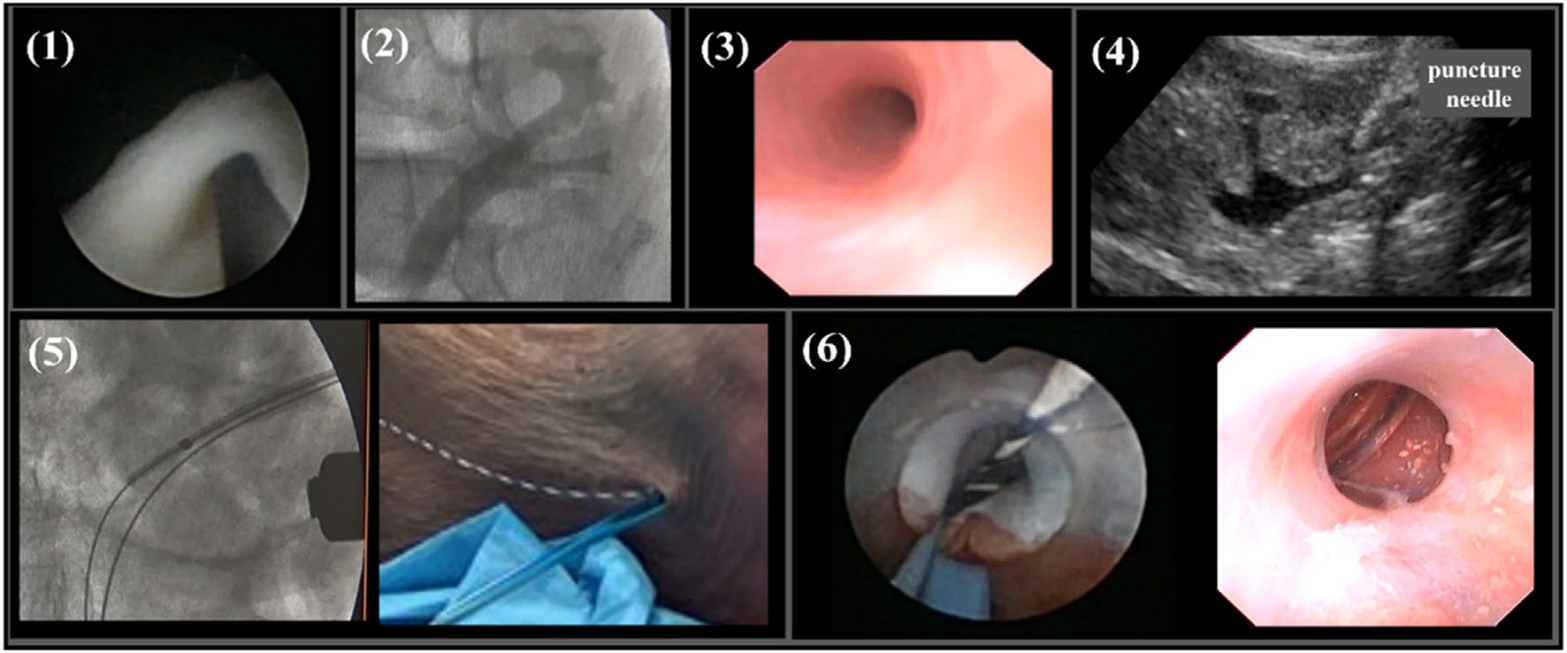
Surgical steps of ECIRS in a fresh‐frozen cadaver. (1) Insertion of a guidewire into the ureter. (2) Retrograde pyelography. (3) Observation of the ureter via flexible ureteroscope. (4) Ultrasound‐guided nephrostomy puncture. (5) Nephrostomy tract creation. (6) Observation of the renal pelvis via pyeloscopy.

#### 2.3.2 Second Period (April 2022–October 2022)

During the second period, laparoscopic nephrectomy CST preceded ECIRS‐CST using the same fresh‐frozen cadavers. Laparoscopic nephrectomy CST was performed in the standard flank position, after which the cadaver was repositioned to the modified Valdivia position for ECIRS‐CST. During laparoscopic training, the renal vein was clipped with Hem‐o‐lok clips to prevent venous backflow from the urinary tract (Figure 2).

**FIGURE 2:**
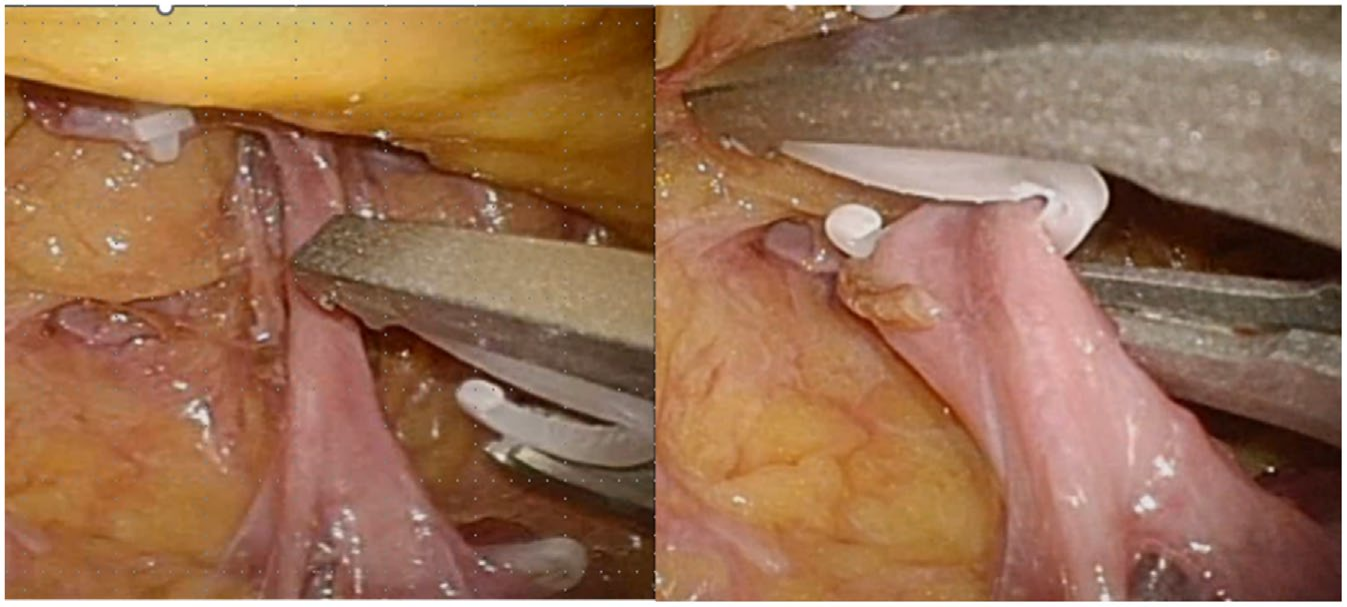
Clipping of renal vein in a fresh‐frozen cadaver.

The ureter was dissected distally and temporarily exteriorized through a 5‐mm port to create temporary ureterocutaneous access, through which a ureteral access sheath was inserted. As a result, cystoscopic guidewire insertion into the renal pelvis (Step 1) was omitted. However, cystoscopy was performed to confirm bladder anatomy and procedural completeness. Steps 2–6 were then carried out as in the first period, including retrograde hydronephrosis creation, ultrasound‐guided nephrostomy puncture, tract dilation, and nephroscopic inspection.

### 2.4 Outcome Measures

#### 2.4.1 Primary Outcome

The primary outcome was successful ultrasound‐guided nephrostomy puncture (Step 4), defined as puncture of the renal pelvis under ultrasound guidance with confirmation of contrast efflux.

#### 2.4.2 Secondary Outcomes

The secondary outcomes were successful completion of all ECIRS steps through nephroscopic inspection (Step 6), the achievement rate of each procedural step, and subjective ratings of procedural realism and educational value using a custom‐designed questionnaire.

### 2.5 Questionnaire Assessment

After each ECIRS‐CST session, the participants completed a custom‐designed 29‐item questionnaire developed with reference to the questionnaire reported by Veys et al. [3]. The questionnaire assessed the realism and educational usefulness of each ECIRS component, including cystoscopy, ureteroscopy, hydronephrosis creation, ultrasound‐guided nephrostomy puncture, tract dilation, and nephroscopic inspection.

Items were rated on a 5‐point Likert scale (5 = strongly agree to 1 = strongly disagree). Higher scores indicated greater similarity to live‐patient surgery and higher educational value.

### 2.6 Statistical Analysis

Data were analyzed using SPSS software (IBM Corp., Armonk, NY, USA). Continuous variables are reported as means. The success rate of ultrasound‐guided nephrostomy puncture was compared between the first and second periods using Fisher's exact test. A two‐sided *p*‐value < 0.05 was considered statistically significant.

This study was approved by the Ethics Committee of Tokushima University (ToCMS ID: 3897).

## 3 Results

### 3.1 Overview of ECIRS‐CST Sessions

During the study period, 13 ECIRS‐CST sessions were conducted using fresh‐frozen cadavers. In the first period, nine sessions were performed using seven cadavers. In the second period, four sessions were conducted using three cadavers.

### 3.2 Primary Outcome: Success of Nephrostomy Puncture

Successful ultrasound‐guided nephrostomy puncture (Step 4) was achieved in three of nine sessions (33%) in the first period and three of four sessions (75%) in the second period.

Although the success rate was higher in the second period, the difference was not statistically significant (Fisher's exact test, *p* = 0.266).

### 3.3 Achievement of ECIRS Procedural Steps

#### 3.3.1 First Period

In the first period, ECIRS‐CST was performed without renal vein clipping. Among the nine sessions, guidewire insertion into the ureter (Step 1) was not achieved in three sessions because the ureteral orifice could not be clearly identified. In three additional sessions, ultrasound‐guided nephrostomy puncture (Step 4) was unsuccessful, likely due to difficulty in achieving sufficient hydronephrosis, as suggested by retrograde pyelography findings (Figure 3). Consequently, only three of nine sessions progressed through all ECIRS steps to nephroscopic inspection of the renal pelvis (Step 6).

**FIGURE 3:**
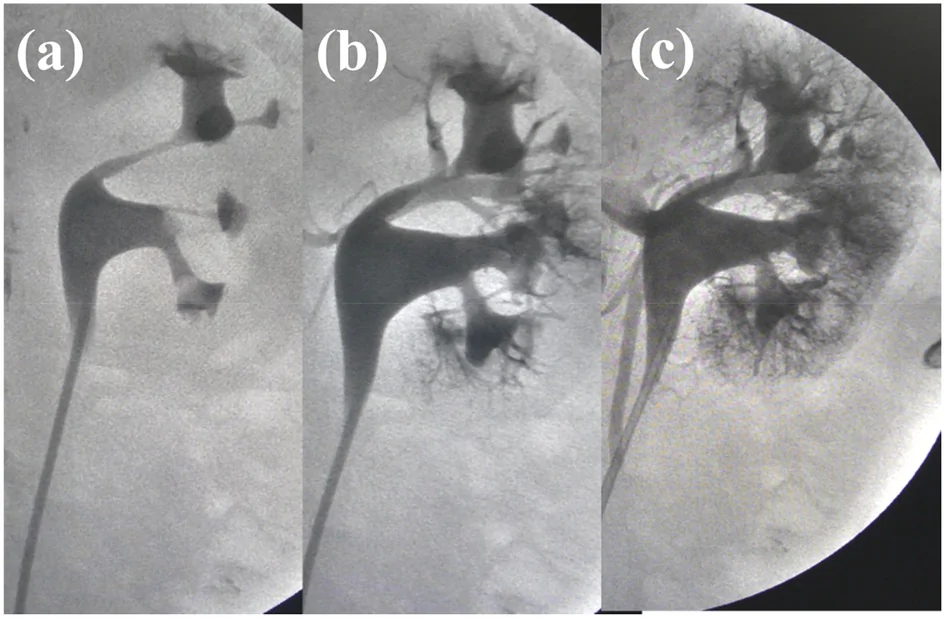
Retrograde pyelography demonstrating intrarenal and pyelovenous backflow in a fresh‐frozen cadaver. (a) Early phase showing minimal pelvic dilatation. (b) Intermediate phase demonstrating reflux of contrast medium into the venous system. (c) Late phase showing extensive venous opacification with insufficient pelvic expansion.

#### 3.3.2 Second Period

In the second period, laparoscopic nephrectomy training with renal vein clipping preceded ECIRS‐CST. Of four sessions, ultrasound‐guided nephrostomy puncture (Step 4) failed in one. This failure occurred after thoracic surgical training had been performed on the same cadaver, resulting in cranial displacement of the kidney, which could not be adequately visualized by ultrasonography despite stable hydronephrosis confirmed by retrograde pyelography. Consequently, ultrasound‐guided puncture was technically impossible. In the remaining three sessions, all ECIRS steps were completed through Step 6. Despite the puncture failure in one session, retrograde pyelography confirmed stable hydronephrosis without venous backflow in all four sessions (Figure 4). The achievement of each ECIRS procedural step across all training sessions is summarized in Table S1.

**FIGURE 4:**
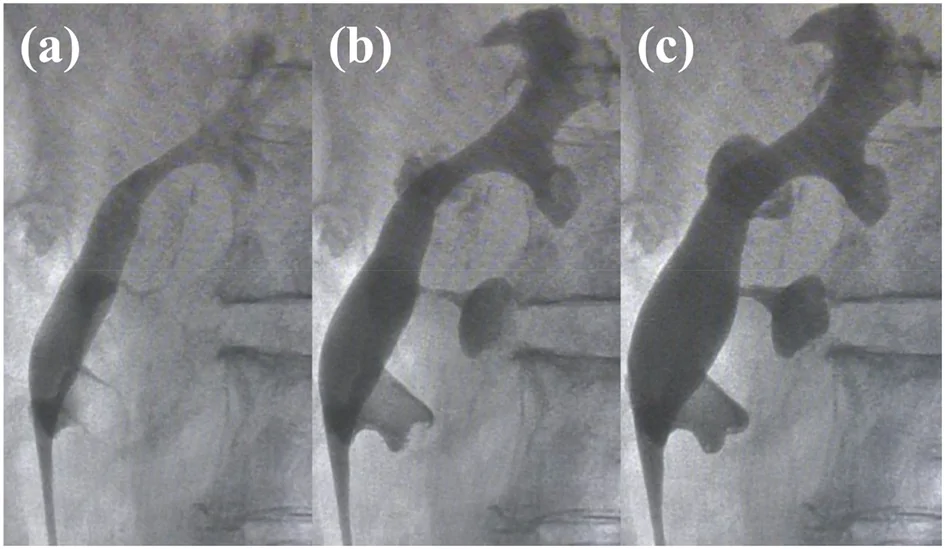
Retrograde pyelography after renal vein clipping in a fresh‐frozen cadaver. (a) Early phase showing initial opacification of the renal pelvis. (b) Intermediate phase demonstrating progressive pelvic and calyceal dilatation without venous reflux. (c) Late phase showing sufficient hydronephrosis for nephrostomy puncture.

### 3.4 Questionnaire‐Based Evaluation of Procedural Realism and Educational Usefulness

All participants completed the 29‐item custom‐designed questionnaire assessing realism and educational value using a 5‐point Likert scale (Figure 5).

**FIGURE 5:**
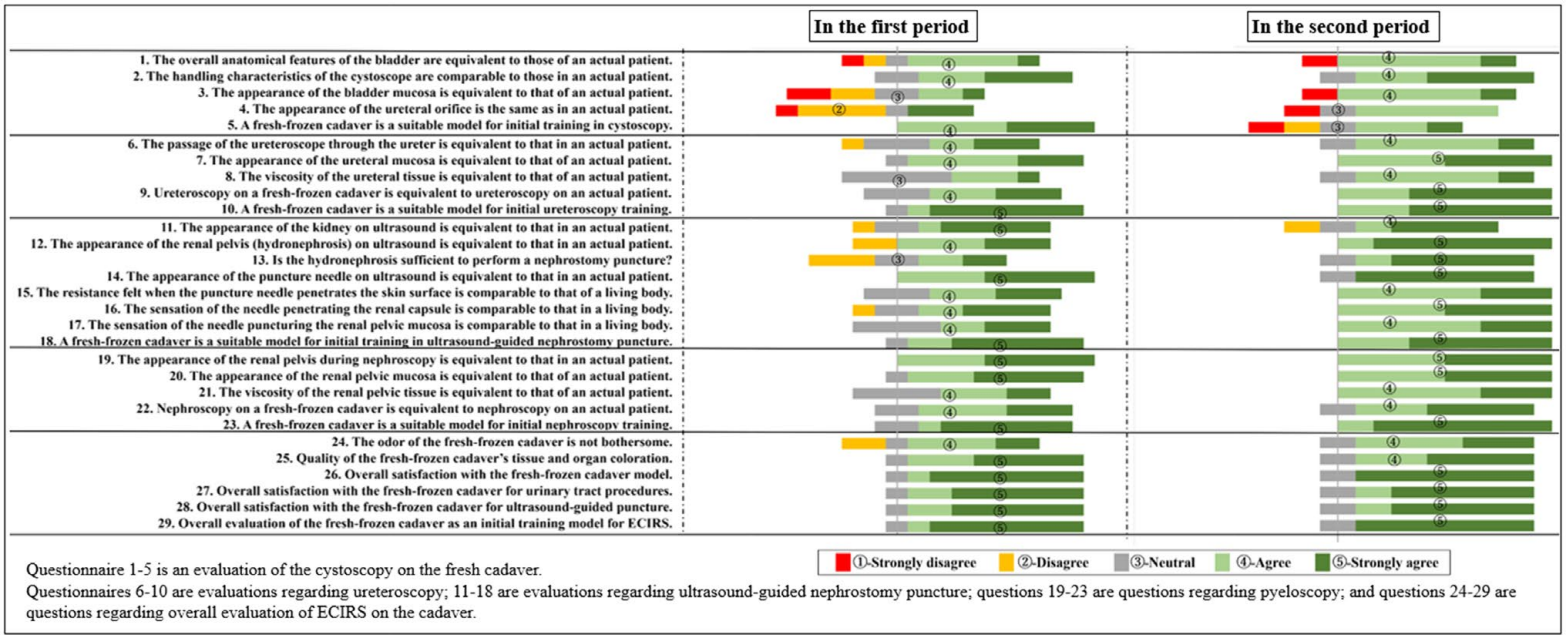
Questionnaire‐based evaluation of ECIRS‐CST using fresh‐frozen cadavers. Results are shown for the first and second periods. Items were rated on a 5‐point Likert scale (1 = strongly disagree to 5 = strongly agree). Questions 1–5 assess cystoscopy, 6–10 ureteroscopy, 11–18 ultrasound‐guided nephrostomy puncture, 19–23 pyeloscopy, and 24–29 overall evaluation of ECIRS.

The responses indicated that the intravesical environment of fresh‐frozen cadavers was less comparable to live patients. In particular, bladder mucosal appearance and ureteral orifice visibility received lower ratings than other components, and identification of the ureteral orifices during cystoscopy was occasionally difficult. For cystoscopy‐related items (Questions 1–5), overall bladder anatomy and cystoscope handling were generally rated as agree or strongly agree in both periods, whereas bladder mucosal appearance and ureteral orifice visibility received lower scores.

For ureteroscopy‐related items (Questions 6–10), passage of the ureteroscope, ureteral mucosal appearance and viscosity, and suitability for initial ureteroscopy training were rated as comparable to live‐patient surgery in both periods.

For ultrasound‐guided nephrostomy puncture (Questions 11–18), kidney appearance on ultrasonography, needle visualization, resistance during penetration of the skin and renal capsule, and sensation of puncturing the renal pelvic mucosa were consistently rated as comparable to live patients in both periods. By contrast, adequacy of hydronephrosis for puncture (Question 13) was rated lower in the first period, reflecting difficulty creating and maintaining sufficient pelvic dilatation. After renal vein clipping in the second period, ratings for hydronephrosis improved, with higher agreement scores for Question 13, indicating more consistent achievement of adequate dilatation. Improved ultrasonographic visualization of the renal pelvis facilitated nephrostomy puncture.

For nephroscopy‐related items (Questions 19–23), visualization of the renal pelvis was generally rated as comparable to live surgery. However, the appearance and viscosity of the renal pelvic mucosa received lower ratings, suggesting increased mucosal fragility in unfixed fresh‐frozen cadavers.

Overall evaluation items (Questions 24–29) showed high agreement in both periods, including overall satisfaction with the fresh‐frozen cadaver model and its suitability as an initial training model for ECIRS and ultrasound‐guided nephrostomy puncture.

## 4 Discussion

The present study evaluated a training model for ECIRS, including nephrostomy puncture, using fresh‐frozen cadavers. Although several studies have demonstrated the usefulness of cadaver‐based ECIRS training with Thiel‐embalmed cadavers, systematic evaluations of ECIRS training that include nephrostomy puncture using fresh‐frozen cadavers are limited, with only one such report to date [3, 6]. That study also noted limitations in ultrasound image visibility in fresh‐frozen cadavers, suggesting potential constraints for ultrasound‐guided nephrostomy puncture in this setting.

By contrast, our findings show that ultrasound‐guided nephrostomy puncture is feasible when adequate hydronephrosis is achieved through renal vein clipping. This modification enhances the practicality of the fresh‐frozen cadaver model for ECIRS training and represents a key contribution of the present study.

Simulation‐based training is now integral to urologic education and includes dry lab models, virtual reality (VR) simulators, animal models, and cadaver‐based training. Dry lab and VR simulators are effective for learning procedural workflows and basic skills but cannot fully replicate tissue characteristics [7, 8, 9]. Animal models provide a living environment but differ anatomically from humans and raise ethical and logistical concerns [10]. Cadaver‐based training, by contrast, faithfully reproduces human anatomy. Thiel‐embalmed cadavers maintain tissue flexibility and color and have demonstrated good face and content validity for endoscopic and laparoscopic urologic training [4, 11, 12]. Fresh‐frozen cadavers have recently gained attention because they more closely preserve tissue texture and haptic feedback [5, 13]. However, despite their realism and preservation of tissue planes, they have practical drawbacks, including limited usable time, infection risk, and higher cost [13, 14].

Multimodal simulation curricula that combine dry lab, VR, and cadaver training have been shown to improve ureteroscopic skills, yet their impact on nephrostomy puncture‐based procedures remains uncertain, highlighting the need for more realistic models [7, 8, 9]. In this context, fresh‐frozen cadavers may provide educational advantages that complement high‐fidelity synthetic simulators. They allow residents to understand the native anatomy of the renal pelvis, calyces, and ureter, practice the entire ECIRS workflow in a single integrated procedure, and develop the spatial awareness required for simultaneous retrograde and percutaneous approaches. Therefore, fresh‐frozen cadaver‐based training may be valuable for acquiring advanced skills required for ECIRS and percutaneous nephrolithotomy (PCNL).

The learning curve for PCNL is reported to range from approximately 30–60 cases when operative time and postoperative complications are considered [2, 15, 16]. When focusing specifically on nephrostomy puncture, however, reported case numbers required for proficiency vary widely from 20 to 120, underscoring the technical difficulty of this step [2].

More recently, Yanase et al. reported that urology trainees required approximately 16–20 ECIRS cases to achieve acceptable surgical outcomes and renal access proficiency under supervised training, suggesting that the combined retrograde–antegrade approach may facilitate acquisition of percutaneous access skills [1]. Achieving this experience may be challenging in low‐volume centers; therefore, realistic simulation platforms may accelerate skill acquisition and improve procedural safety.

CST helps address this training gap by allowing multiple surgeons to practice nephrostomy puncture in a near–live surgical setting within a single session.

Our results indicate that fresh‐frozen cadavers are useful for ECIRS training, although several differences from live surgery were observed. Previous studies of percutaneous renal procedures in fresh‐frozen cadavers have reported technical limitations related to imaging, including reduced ultrasonographic visibility in certain situations, which may compromise reliable ultrasound‐guided access [6]. In addition, the intravesical environment often exhibits low contrast, making ureteral orifice identification difficult; this has also been described with Thiel‐embalmed cadavers [4, 12].

Indeed, three of nine sessions in the first period failed at Step 1 because guidewire insertion through the ureteral orifice was unsuccessful. In the second period, temporary ureterocutaneous access bypassed this step, allowing all sessions to proceed to retrograde hydronephrosis creation. Incomplete bladder thawing and cranial displacement of the kidney due to the absence of respiratory motion may further alter procedural conditions, sometimes necessitating simple adjustments such as warm saline irrigation or slight head‐up positioning.

### 4.1 Hydronephrosis Creation and Intrarenal/Pyelovenous Backflow

A likely explanation for the difficulty in generating sufficient hydronephrosis during the first period is intrarenal backflow, particularly pyelovenous backflow. Intrarenal backflow is a recognized pressure‐relief mechanism in which urine or irrigation fluid escapes from the collecting system into the renal tubules, venous circulation, or lymphatics in response to increased intrapelvic pressure, thereby protecting the renal parenchyma [17]. Traditionally, this phenomenon was thought to occur only at high intrapelvic pressures; however, experimental data suggest that intrarenal backflow can occur even under relatively low‐pressure conditions in a time‐dependent manner [18].

In fresh‐frozen cadavers, the loss of vascular smooth muscle tone and absence of physiologic renal venous pressure likely create a low‐resistance pathway within the venous system when intrapelvic pressure rises, facilitating pyelovenous backflow. As a result, retrograde pyelography or irrigation may fail to maintain sufficient intrapelvic pressure to achieve stable hydronephrosis.

Clinical observations support this mechanism. Even in complete ureteral obstruction, pyelovenous backflow has been reported to decompress the renal pelvis, resulting in only mild hydronephrosis [19]. Thus, the degree of hydronephrosis does not necessarily correlate with the severity of obstruction, and venous backflow may limit pelvic dilatation.

In the first period of our study, retrograde pyelography produced only mild dilation, corresponding to approximately Grade 1 hydronephrosis. This finding is consistent with insufficient intrapelvic pressure due to venous backflow in fresh‐frozen cadavers (Figure 3). After introducing renal vein clipping before ECIRS‐CST, stable hydronephrosis was achieved, with improved nephrostomy puncture success and procedural completion (Figure 4). These results suggest that control of venous backflow is essential for effective ECIRS training using this model. The success rate of ultrasound‐guided nephrostomy puncture increased from 33% (3/9) in the first period to 75% (3/4) in the second period, although the difference did not reach statistical significance. This finding should be interpreted in the context of the small sample size and limited statistical power of the present study. The single unsuccessful case occurred after thoracic surgical training on the same cadaver, resulting in marked cranial displacement of the kidney, which was considered the primary cause of failure.

Overall, fresh‐frozen cadavers provided a realistic environment for most ECIRS steps. Previous studies using Thiel‐embalmed cadavers have reported a firmer tactile sensation when penetrating the skin compared with live patients [4, 12]. In our study, the tactile feedback during penetration of both the skin and renal capsule in fresh‐frozen cadavers was rated as comparable to live surgery. This represents an important advantage of fresh‐frozen cadavers for nephrostomy puncture training [6, 13].

Another limitation is the absence of respiratory motion and tissue perfusion, preventing trainees from experiencing kidney movement or managing puncture‐related bleeding. Perfused fresh cadaver models with mechanical ventilation can reproduce perfusion, bleeding, and respiratory motion, providing higher‐fidelity simulation but require additional equipment and expertise [14]. The present model therefore provides a practical platform for learning fundamental ECIRS skills, whereas adaptation to respiratory motion and bleeding management should be acquired during subsequent supervised clinical practice, bridging simulation‐based training and live‐patient surgery.

The present ECIRS training model offers value not only because multiple trainees can practice nephrostomy puncture in a single session, but also because it enables simulation of the entire ECIRS procedure. Integration of this model with dry lab and VR‐based training may further enhance technical skill acquisition, consistent with structured simulation curricula in urology [7, 8].

Several limitations should be acknowledged. First, because intrarenal pressure was not measured in the present study and renal vein clipping eliminates a physiological pressure‐relief pathway, the pressure environment after renal vein clipping may not reproduce physiological intrarenal pressure in live patients. Therefore, at present, this model is intended primarily to facilitate procedural training by enabling stable hydronephrosis rather than to reproduce physiological intrarenal pressure conditions. However, beyond education, the model may also support research applications, such as investigation of intrarenal pressure during endoscopic procedures and optimization of irrigation strategies [17, 18, 20]. Incorporation of pressure sensors and quantitative monitoring of irrigation volume may enable objective assessment of pressure dynamics in future studies. Such developments could expand the utility of this model to include research and education in safe intrarenal pressure management. Second, we did not directly compare fresh‐frozen cadavers with other models, such as Thiel‐embalmed or formalin‐fixed specimens [4, 11, 12]. Third, the custom‐designed questionnaire, developed with reference to a previously published questionnaire for cadaveric ECIRS training, was not psychometrically validated and provided subjective assessments. Objective procedural metrics, including puncture time, number of attempts, fluoroscopy time, and accuracy of calyceal access, were also not prospectively recorded. Future studies should incorporate validated assessment tools and objective performance metrics. Fourth, the sequential study design may have introduced a learning‐curve effect contributing to the improved puncture success in the second period, although participants differed across sessions. Fifth, the extent to which repeated endourological procedures can be performed on a single fresh‐frozen cadaver remains uncertain [13, 14]. Because cadaveric ureteral and renal tissues lack normal muscle tone, prior dilation of the ureter or renal tract during an initial session may influence subsequent training conditions. This issue warrants further investigation.

In conclusion, fresh‐frozen cadavers provide a realistic training environment for ECIRS, including ultrasound‐guided nephrostomy puncture. However, inadequate hydronephrosis is a major limitation. Renal vein clipping was associated with stable hydronephrosis and higher observed rates of nephrostomy puncture success and procedural completion in this small feasibility study. Additional procedural modifications, such as temporary ureterocutaneous access, may further facilitate training in selected settings.

## Author Contributions

**Keito Shiozaki:** conceptualization, methodology, writing – original draft, investigation, data curation, funding acquisition, writing – review and editing, visualization, formal analysis. **Yutaro Sasaki:** investigation, funding acquisition. **Katsuhito Hori:** investigation. **Marika Atagi:** investigation. **Ryota Nakanishi:** investigation. **Keisuke Hashimoto:** investigation. **Koki Tada:** investigation. **Fumiya Kadoriku:** investigation. **Takuya Yoshioka:** investigation. **Kyotaro Fukuta:** software. **Yuichiro Atagi:** visualization. **Saki Kobayashi:** investigation. **Ryoei Minato:** formal analysis. **Ryotaro Tomida:** writing – review and editing. **Yoshito Kusuhara:** writing – review and editing. **Kunihisa Yamaguchi:** visualization. **Tomoya Fukawa:** writing – review and editing. **Hirofumi Izaki:** supervision. **Koichi Tomita:** resources. **Junya Furukawa:** writing – review and editing.

## Funding

This study was funded by the Japanese Society of Endourology and Robotics (JSER) under the CST Education and Training Grant programs in 2021, 2022, and 2023.

## Ethics Statement

The study was approved by the Ethics Committee of Tokushima University (ToCMS ID: 3897). All cadavers were donated to Tokushima University for medical education and research through an institutional body donation program. All procedures complied with institutional regulations and the Declaration of Helsinki for research involving human materials.

## Consent

Written informed consent was obtained from the donors and/or their families before death.

## Conflicts of Interest

Junya Furukawa is an Editorial Board member of the *International Journal of Urology* and a coauthor of this article. To minimize bias, they were excluded from all editorial decision‐making related to the acceptance of this article for publication. All other authors do not have any conflicts of interest.

## Supporting information


**Table S1:** Achievement of procedural steps in each ECIRS training session. 〇 = Successful. × = Failed. – = Not attempted because the preceding step was unsuccessful. Step 1: Insertion of a guidewire into the ureter. Step 2: Placement of a ureteral access sheath. Step 3: Creation of hydronephrosis by retrograde pyelography. Step 4: Ultrasound‐guided nephrostomy puncture. Step 5: Nephrostomy tract creation. Step 6: Observation of the renal pelvis via rigid nephroscope. *Step 1 was omitted in the second period because guidewire placement had already been completed during the preceding laparoscopic nephrectomy training. Participants are presented as total participants (number of residents). Two board‐certified urologists participated in all sessions.

## Data Availability

For all reports containing original data, the principal investigator had full access to all the data in the study and takes responsibility for the integrity of the data and the accuracy of the data analysis. The datasets generated and/or analyzed during the current study are available from the corresponding author on reasonable request.
